# Highly Robust and Selective System for Water Pollutants Removal: How to Transform a Traditional Photocatalyst into a Highly Robust and Selective System for Water Pollutants Removal

**DOI:** 10.3390/nano9111509

**Published:** 2019-10-23

**Authors:** Olga Sacco, Vincenzo Vaiano, Christophe Daniel, Wanda Navarra, Vincenzo Venditto

**Affiliations:** 1 Dipartimento di Chimica e Biologia and INSTM Research Unit, Università degli Studi di Salerno, Via Giovanni Paolo II 132, 84084 Fisciano, Italy; osacco@unisa.it (O.S.); wnavarra@unisa.it (W.N.); vvenditto@unisa.it (V.V.); 2 Department of Industrial Engineering, The University of Salerno, Via Giovanni Paolo II 132, 84084 Fisciano, Italy

**Keywords:** ZnO, aerogels, photocatalyst support, water pollutants photodegradation, nanoporous crystalline phases

## Abstract

Highly porous monolithic aerogels based on ZnO photocatalyst and syndiotactic polystyrene (s-PS) were obtained by supercritical CO_2_ treatment of ZnO/s-PS gels. The prepared aerogels were characterized and their photocatalytic activity was evaluated using phenol and toluene as water pollutant models. The s-PS nanoporous crystalline phase, able to absorb pollutant molecules, was proven to be necessary to ensure high photocatalytic efficiency as the aerogel acts not only as a support, but also as pollutant pre-concentrator. The reusability of ZnO/s-PS aerogels is also strong showing no decrease in photocatalytic activity after six consecutive degradation trials. Finally, the aerogel matrix prevents ZnO dissolution occurring under acidic conditions and promotes a selective removal of the pollutants. The synergy between the photocatalyst and the innovative polymeric support provides the composite system with robustness, chemical stability, easy recovery after treatment, high efficiency of pollutant removal with a marked selectivity which make these materials promising for large scale applications.

## 1. Introduction

Persistent organic pollutants (POPs), commonly non-biodegradable toxic compounds, are a major issue for water pollution and a large number of processes for removing POPs from wastewater, surface water as well as subsurface water, have been proposed. Sorption-based processes are among the most frequently used conventional technologies for water treatment. The activated carbons constitute the more common class of absorbent, although new classes, such as resins, zeolites, clays, and natural biosorbents, are continually tested and proposed [1,2].

However, all absorbent classes have the obvious drawback that safe handling and disposal of adsorbents containing pollutants entail additional costs. Therefore, many efforts are still underway to develop sustainable and cost-efficient treatment processes and new technologies, such as biodegradation and advanced oxidation processes (AOPs), which have been proposed to effectively remove or degrade toxic organic pollutants from water. Biological treatments are the most economical ones, but present limited efficiency for the removal of POPs and produce sludge, while AOPs, such as Fenton like processes or ozonation, are characterized by high operating costs (chemicals and energy consumption). On the other hand, photocatalytic degradation process of POPs has instead gained much attention due to its effectiveness in fast degradation and mineralization of recalcitrant organic compounds, and many metal-oxide photocatalysts, such as TiO_2_, SnO_2_, and ZnO, have being extensively studied [3,4].

An important drawback of the photodegradation process is the post-treatment recovery of phototocatalyst nanoparticles from the treated water which is both time and money consuming. In order to overcome this limitation, a large variety of possible inorganic and organic supports such as glass mats, natural and synthetic fabrics, polymers, clays, zeolites, etc. have been tried for supporting photocatalyst nanoparticles [5].

In addition to an easy nanoparticles (NPs) recovery, the ideal NPs/support must also meet the following features:

(1) Permanent immobilization of NPs on or within the support.
(2) No decrease of the photocatalyst NPs efficiency.
(3) Good chemical and mechanical stability.

Among the potential substrates, polymers are particularly interesting as they are chemically inert and mechanically stable with high durability [6,7,8,9,10], inexpensive and readily available, and hydrophobic. Thus, polymers are capable of pre-concentrating the organic pollutants, making them floatable and therefore easy to recover. However, in order to minimize the loss of the photocatalyst, NPs are generally completely or partly embedded in a polymer matrix therefore leading to a lower photocatalytic efficiency of the immobilized photocatalysts with respect to the water dispersed NPs [6].

Among the polymer supports, high porosity polymeric aerogels are especially appealing as they offer many benefits over typical polymeric supports, such as high surface area and sorption capacity and many examples of aerogel photocatalysts, have been reported in literature [7,8]. These aerogels can be obtained either by the direct synthesis of aerogel photocatalysts by a sol-gel process or by the incorporation of the photocatalytic moieties within the aerogel 3D network. While the former aerogels usually suffer from tedious preparation and poor mechanical properties, composite aerogels present excellent mechanical properties and are easier to prepare [8].

It has been recently shown that photocatalysts N-doped TiO_2_ (NdT) dispersed in syndiotactic polystyrene (s-PS) monolithic aerogels presents a higher phenol degradation efficiency than NdT powders [9]. This result was mainly attributed to an efficient dispersion of NdT nanoparticles in the composite aerogels, thus avoiding the aggregation that typically occurs when photocatalyst NdT powders are used in slurry reactors.

In this paper, the use of polymeric aerogels as a support of photocatalyst nanoparticles has been extended to ZnO. In the last decade, ZnO, which is one of the most promising semiconductors, has received much attention because it has been found to be more efficient than TiO_2_ for the photodegradation of organic pollutants [11,12]. Moreover, ZnO is less toxic and cheaper than TiO_2_.

The photodegradation efficiency and effective reusability of monolithic composite aerogels based on ZnO photocatalyst and s-PS has been evaluated using phenol and toluene as water pollutant models. Both these POP pollutants, frequently found in the environment due to their widespread use, are listed as priority toxic pollutants by the United States Environmental Protection Agency. The maximum concentration limits for them are listed in many drinking water and wastewater discharge regulations and for instance, the wastewater discharge limit into surface waters set by the Italian Environmental Protection Code (Legislative Decree 152/2006) is 0.5 mg/L for phenol (listed as phenols) and 0.2 mg/L for toluene (listed as organic aromatic solvents).

The results reported here clearly show that the photodegradation efficiency and selectivity of ZnO/s-PS composite aerogels strongly depend on the polymer crystal structure and its affinity towards non-polar organic compounds. Moreover, the excellent mechanical properties and chemical resistance of polymeric matrix also prevents the ZnO dissolution in strongly acidic or basic solutions, allowing an easy recovery of ZnO/s-PS aerogels after water treatment and extending the condition range of its practical use. Based on these results, the contributions of the s-PS polymer matrix and ZnO photocatalyst to the composite system behavior can be properly considered synergic.

## 2. Materials and Methods

### 2.1. Materials

The syndiotactic polystyrene (s-PS) used for aerogels preparation was manufactured by Idemitsu Kosan Co., Ltd. (Tokyo, Japan) under the trademark XAREC© 90ZC. The polymer was highly stereoregular having a content of syndiotactic triads over 98% (^13^C nuclear magnetic resonance data). The solvents, reagents and zinc oxide (ZnO) particles were purchased from Sigma-Aldrich (Saint Louis, MO, USA) and used without further purification.

### 2.2. Preparation of ZnO Nanocomposite Aerogels

All s-PS/ZnO gel samples were prepared, by using chloroform as a solvent, in hermetically sealed test tubes by heating the mixtures above the boiling point of chloroform until the complete dissolution of the polymer and the appearance of a transparent and homogeneous solution had occurred. Then, the hot solutions were cooled down to room temperature under sonification with a Bandelin Sonorex instrument (RK 1028 H, Berlin, Germany) until gelation occurred. Then, the obtained gels were treated with carbon dioxide (CO_2_) at supercritical condition (by using an ISCO SFX 220 extractor, Lincoln, NE, USA) to extract the solvent and obtain the relative monolithic composite aerogels with a cylindrical shape (d = 0.5 cm, h = 10 cm). The ZnO/s-PS aerogels with the s-PS δ-crystalline form was obtained after extraction at T = 40 °C, P = 19,994.8 kPa and an extraction time of 4 h, while the ZnO/s-PS aerogels with the s-PS δ-crystalline form were obtained after extraction at T = 120 °C, P = 19,994.8 kPa and an extraction time of 4 h.

The ZnO/s-PS weight ratio in the aerogel samples, labelled as 2/5/15 ZnO/s-PS, was 2/98, 5/95, 15/85, respectively, while the solvent/s-PS weight ratio was 90/10 for all samples.

### 2.3. Samples Characterization

The catalysts were characterized by several techniques. The UV-vis reflectance spectra of catalysts were recorded by a Perkin-Elmer spectrophotometer Lambda 35 using a RSA-PE-20 reflectance spectroscopy accessory (Labsphere Inc., North Sutton, NH, USA). All spectra were obtained using an 8° sample positioning holder, giving the total reflectance relative to a calibrated standard SRS-010-99 (Labsphere Inc., North Sutton, NH, USA).

The specific surface area of the ZnO powder and aerogel samples, determined by the BET method, was obtained by dynamic N_2_ adsorption measurements using a Nova Quantachrome 4200e instrument analyzer (Boyton Beach, FL, USA).

The wide-angle X-ray diffraction (WAXD) patterns were performed with an automatic Bruker D8 Advance diffractometer (VANTEC-1 detector) using reflection geometry and nickel filtered Cu-Kα radiation. The intensities of WAXD patterns were not corrected for polarization and Lorentz factors, to allow for an easier comparison with most of the literature data.

The internal morphology of the monolithic aerogel was characterized by means of a scanning electron microscope (SEM, Zeiss Evo50, Jena, Germany). equipped with an Oxford energy dispersive X-ray detector). The samples were prepared by fracturing in liquid N_2_ small pieces of the monoliths in order to gain access to the internal part of the sample. Before imaging, all the specimens were coated with gold using a VCR high resolution indirect ion-beam sputtering system. The samples were coated depositing approximately 20 nm of gold. The coating procedure was necessary in order to prevent surface charging during the measurement and to increase the image resolution.

### 2.4. Photocatalytic Activity Tests

Phenol was used as a model pollutant: It is a real pollutant, stable under irradiation (no photolysis) and its intermediate degradation products are well known [13]. The initial concentration of phenol was equal to 50 mg/L, at ambient temperature and pressure. Other photocatalytic tests were also conducted using toluene (as additional model pollutant) at a concentration equal to 50 mg/L. For both cases, the spontaneous pH of the solution was equal to 6.7 and did not change significantly during the whole reaction time. For photocatalytic tests in the range 2–8, the pH was adjusted to a given value by the addition of HCl (1 N) or NaOH (1 N) aqueous solutions.

All photocatalytic activity tests were carried out with 75 mL of aqueous solutions and 300 mg of ZnO/s-PS aerogel with ZnO content from 2 wt% (corresponding to 0.08 g/L of ZnO with respect to the treated aqueous solution) to 15 wt% (corresponding to 0.6 g/L of ZnO with respect to the treated aqueous solution), while for the photocatalyst test with neat ZnO powder, a photocatalyst dosage equal to 0.08 g/L (with respect to the treated aqueous solution) was used.

The experiments were realized using a pyrex cylindrical photoreactor equipped with an air distributor device (Q_air_ = 150 cm^3^/min (STP)). The continuous mixing of the wastewater was realized by the external recirculation of wastewater through the use of a peristaltic pump. The thermocouple was inserted inside the reactor to monitor the temperature during irradiation. The photoreactor was irradiated with four UVA lamps (provided by Philips; nominal power: 8 W each) with the main wavelength emission at 365 nm. The system was left in the dark for 120 min to reach the adsorption equilibrium of the target pollutants and then photocatalytic reaction was carried out for 180 min. The variation of pollutant concentration was measured with a Perkin Elmer UV-Vis spectrophotometer at λ = 270 and 261 nm, for phenol [14] and toluene [15], respectively. A standard calibration curve was obtained for different concentrations and allowed to convert absorbance to concentration (mg/L) units. The photocatalytic activity was also tested in terms of the total organic carbon (TOC) reduction that is a parameter able to analyze the mineralization of the tested aromatic compounds. The TOC of the solution was measured from CO_2_ obtained by catalytic combustion at T = 680 °C. CO_2_ produced in the gas-phase was monitored by continuous analyzers, measuring CO, CO_2_ (Uras 14, ABB) and O_2_ (Magnos 106, ABB) gaseous concentrations [14].

## 3. Results and Discussion

The high porosity monolithic polymeric aerogels containing photocatalytic ZnO nanoparticles were prepared by the solvent removal from s-PS/ZnO gels using supercritical carbon dioxide (sc-CO_2_) in order to avoid the gel collapsing. The gels can be easily obtained by heating ZnO and polymer at high temperature in solvents (such as chloroform) able to fully disperse ZnO nanoparticles, dissolve s-PS and give a thermoreversible gel after cooling at room temperature. It is worth noting that the gelling process of s-PS/ZnO/solvent mixture occurs within a few min after cooling at room temperature.

Depending on the supercritical extraction temperature of the solvent present in s-PS gels, aerogels having either a nanoporous or non-porous crystalline phase can be obtained [16].

**Figure 1:**
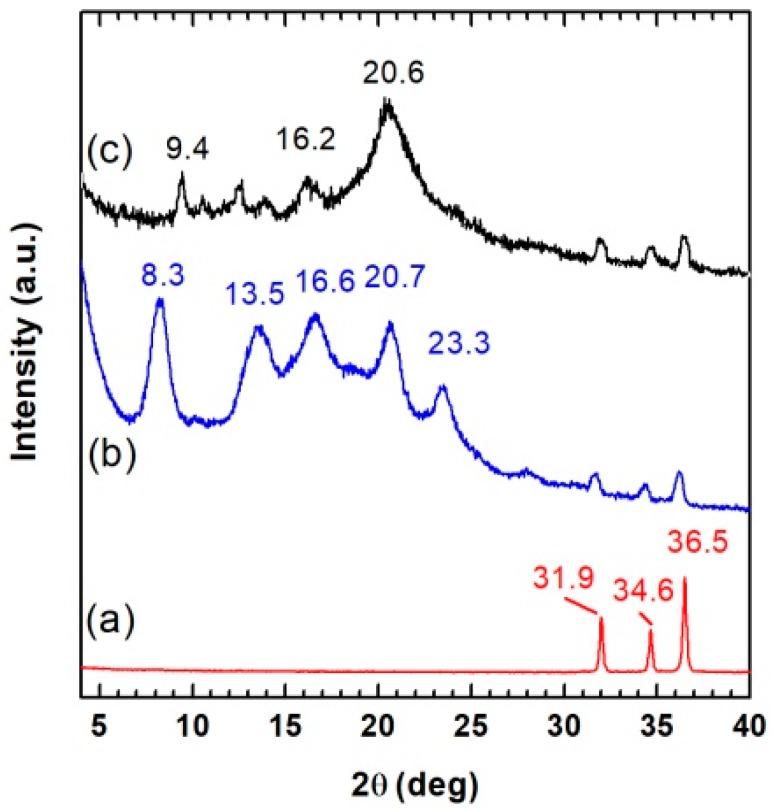
The WAXD patterns of ZnO powder (curve **a**) and of 2ZnO/s-PS aerogels obtained by solvent extraction with sc-CO_2_ at 40 °C (curve **b**) and 120 °C (curve **c**) of gels prepared with a 90/10 solvent/s-PS and 2/98 ZnO/s-PS weight ratio.

The WAXD patterns of neat ZnO powder (curve a) and 2ZnO/s-PS aerogels obtained by solvent extraction at 40 °C (curve b) and 120 °C (curve c) of gels prepared with a 90/10 solvent/s-PS weight ratio and 2/98 ZnO/s-PS weight ratio is shown in Figure 1.

The diffraction patterns of both ZnO/s-PS aerogels (curves b and c) reported in Figure 1 are characterized by diffraction peaks at 2θ = 31.9, 34.5, and 36.5° which are typical of the ZnO wurtzite phase (curve a). However, in the lower diffraction angle range, the diffraction patterns clearly differ depending on the polymer crystalline phase obtained in the aerogel. In particular, the ZnO/s-PS aerogel obtained after extraction at 40 °C (curve b) displays diffraction peaks at 2θ = 8.3, 13.5, 16.6, 20.7 and 23.3° being typical of the nanoporous s-PS δ-form [17] while the ZnO/s-PS aerogel obtained after extraction at 120 °C (curve c) displays diffraction peaks at 2θ = 9.4, 16.2 and 20.6° typical of the non-porous s-PS γ-form [18].

**Figure 2:**
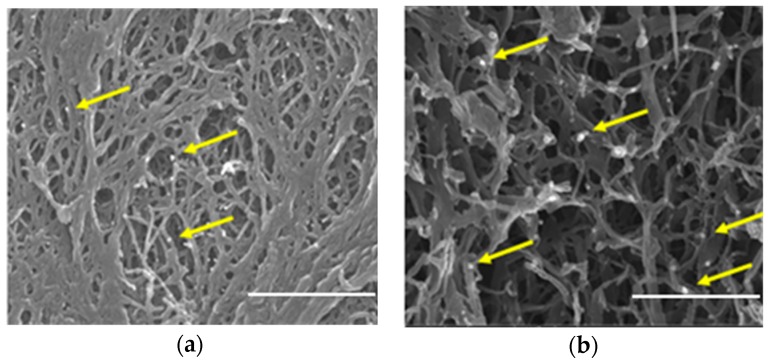
SEM images of 2ZnO/sPS δ (**a**) and γ-form aerogels (**b**). Some ZnO nanoparticles dispersed in the aerogels are indicated by yellow arrows (scale bar: 1 µm).

The SEM images reported in Figure 2 show that both 2ZnO/sPS δ and γ-form aerogels are characterized by a fibrillary 3D network, with a fiber diameter in the range 40–100 nm and pores of a few hundreds of nm, while the ZnO nanoparticles with a size of roughly 10–60 nm (some of them are indicated as an example by yellow arrows) are uniformly dispersed within the fibrillary polymer network.

Although the apparent porosity (Column 3 of Table 1) and morphology (Figure 2) of the two composite aerogels are substantially identical, the surface area of s-PS γ-form based aerogel is 77 m^2^/g, while the s-PS δ-form based one is 276 m^2^/g. This difference in the BET surface area, also observed in the pure s-PS δ and γ-form aerogels [16], is due to the presence of the nanometer-sized pores within the crystalline lattice of the s-PS crystalline δ-form. These pores are absent in the s-PS crystalline γ-form, which therefore has a much smaller surface area [19].

**Figure 3:**
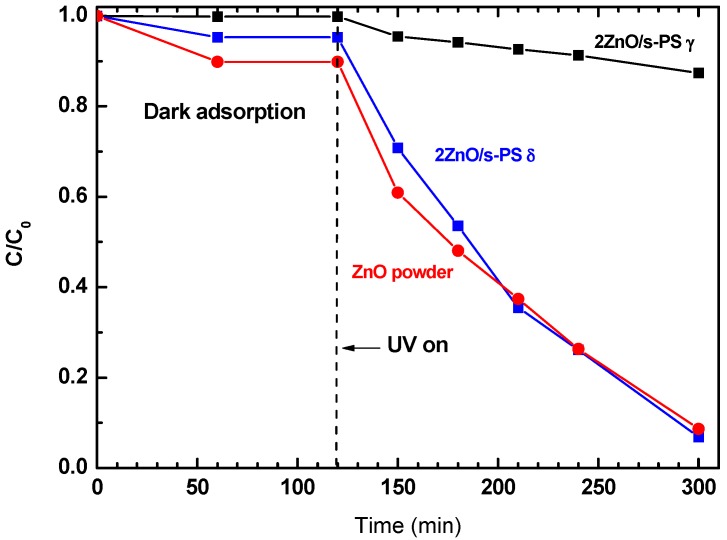
Phenol concentration in aqueous solutions treated with 2ZnO/s-PS δ and γ-form aerogels and ZnO powder. ZnO dosage = 0.08 g/L, phenol C_0_ = 50 mg/L.

The photocatalytic activity of the ZnO powder and 2ZnO/s-PS δ and γ-form aerogels in aqueous solution containing 50 mg/L of phenol were investigated. The phenol degradation results are reported in Figure 3 and summarized in Table 1 (Column 6).

Figure 3 clearly shows that aerogels presenting different s-PS crystalline forms display totally different photocatalytic efficiency. After 180 min of UV irradiation, a phenol aqueous solution exposed to 2ZnO/s-PS γ-form aerogel showed only 13% of phenol degradation, while by using 2ZnO/s-PS δ-form aerogel, phenol degradation was approximately 96%, equal to that obtained by using pure ZnO powder.

A closer look at the phenol degradation curves of Figure 3 shows that during the dark phase (i.e., in the first 120 min) the decrease of phenol concentration is different for the δ- and the γ-form s-PS aerogels. During this initial step, the phenol concentration decrease is only due to the sorption of the phenol molecules within aerogels, which strongly depends on the sorption capacity of the polymer crystalline phases. In detail, after 120 min, using the 2ZnO/s-PS δ-form aerogel, the phenol concentration decreased by approximately 5%. However, while using the 2ZnO/s-PS γ-form aerogel, no phenol concentration decrease was observed. This difference is due to the nanoporous feature of the s-PS crystalline δ-form which is able to absorb phenol molecules into the crystal lattice, while no phenol sorption occurs in the impenetrable non-porous s-PS crystalline γ-form [20].

**Figure 4:**
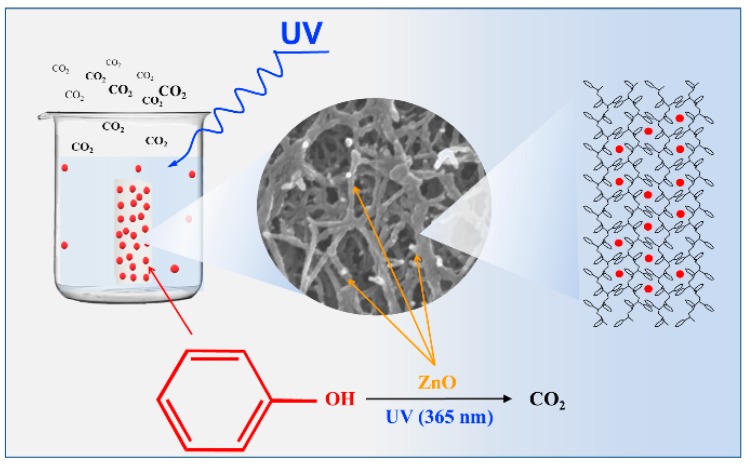
Scheme of the phenol sorption and photocatalysis within the ZnO/s-PS aerogels characterized by the nanoporous δ-form.

In the presence of light irradiation (i.e., after 120 min), the observed difference in photocatalytic activity of the two aerogels clearly shows the primary role of the s-PS polymer crystal structure in the photodegradation process. The phenol degradation process by the ZnO/s-PS δ-form aerogel is sketched in Figure 4.

The following processes, phenol sorption into s-PS nanoporous crystalline δ-form and the UV activated degradation of phenol adsorbed onto ZnO catalytic sites, occur together within the composite aerogel. The aerogel components have a synergistic action in that the crystalline nanoporous phase acts as a pollutant pre-concentrator leading to a highly efficient light-triggered photocatalysis process.

The different photocatalytic activity observed for the two s-PS aerogels clearly shows the primary role of the aerogel structure on the photodegradation process. In particular, the crystalline nanopores, being in the polymer fibrils, acts as a phenol preconcentrator leading to a highly efficient light-triggered photocatalysis process as schematically shown in Figure 4.

**Figure 5:**
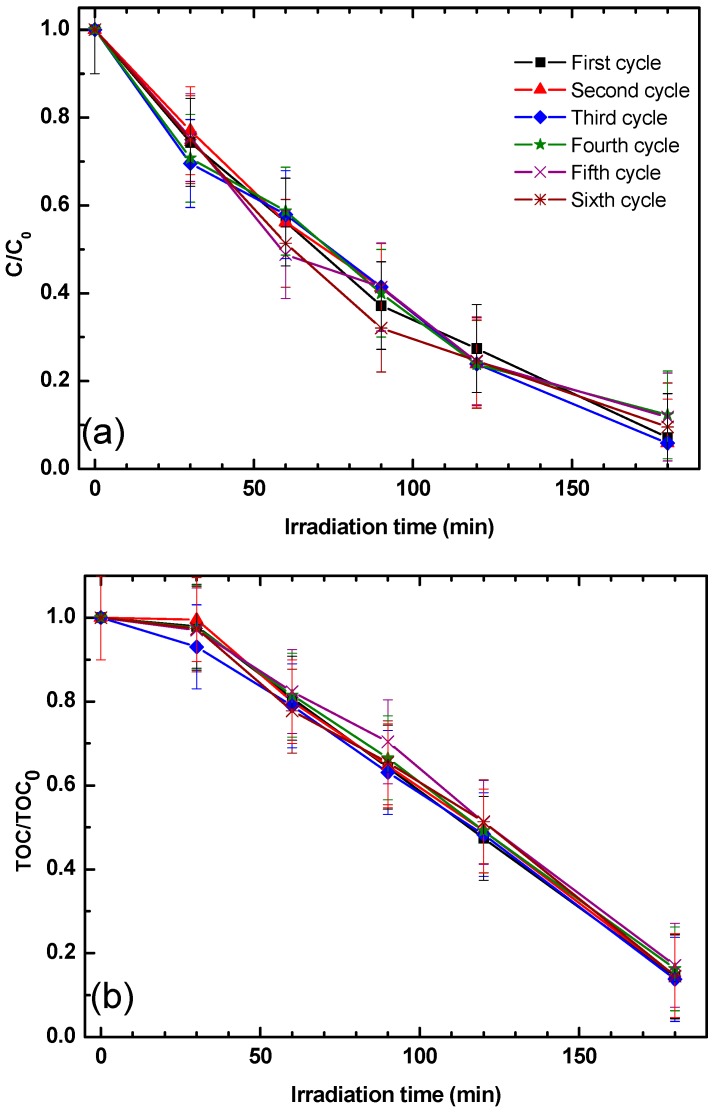
(**a**) phenol and (**b**) the total organic carbon (TOC) concentration aqueous solutions treated with the same 2ZnO/s-PS δ-form aerogel sample. No regeneration step of the aerogel was performed at the end of each cycle.

The reusability and stability of the ZnO/s-PS δ-form aerogel was also investigated and the phenol and total organic carbon (TOC) removal was obtained in six successive cycles using the same experimental conditions and without any regeneration step of the aerogel as shown in Figure 5.

Figure 5a shows that the phenol degradation degree obtained by using the 2ZnO/s-PS δ-form aerogel does not change in all the cycles, showing that the system is stable. This result is in agreement with the one obtained by using N-doped TiO_2_ dispersed in s-PS δ-form aerogels for the photocatalytic degradation of methylene blue dye [10]. Moreover, the results reported in Figure 5b show that 2ZnO/s-PS δ-form aerogel is also able to reduce the TOC after each cycle, always leading to a TOC removal of 86% after 180 min of UV irradiation. The small difference between the final values of phenol degradation and TOC removal was possibly due to the formation, during the degradation processes, of some intermediates, such as hydroquinone, benzoquinone and organic acids, some of which were not readily degradable [21,22,23].

The similar efficiency of pollutant photodegradation after several successive cycles indicates that the ZnO nanoparticles are not released from the aerogel matrix and a full recovery of the aerogel pollutant sorption capacity occurs during the UV light cycle.

The influence of the content of ZnO on the aerogel photocatalytic activity was also investigated and the ZnO/s-PS δ-form aerogels with ZnO NPs loading up to 15 wt% were prepared and characterized.

For the whole range of ZnO concentration being considered, the ZnO wurtzite phase is always obtained and the polymer crystallinity appears substantially constant (X_c_ ≈ 40%) as shown in Figure S1 of Supplementary Materials.

**Figure 6:**
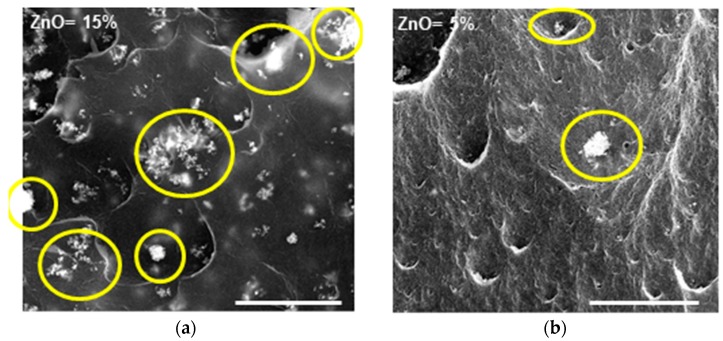
SEM images of the ZnO/s-PS δ-form aerogels with 15 wt% (**a**) and 5 wt% (**b**) of ZnO. Some ZnO aggregates are indicated by yellow circles (scale bar: 50 μm).

In Figure 6, typical SEM images of the ZnO/s-PS aerogels with 15 and 5 wt% of ZnO are shown.

While a uniform dispersion of non-aggregated ZnO nanoparticles was observed for a 2 wt% of ZnO (shown in Figure 2), for higher ZnO amounts, large aggregates sizing from approximately 10 to 30 µm, are formed. Moreover, the SEM images also show that the number of ZnO aggregates increases by increasing the ZnO amount.

**Figure 7:**
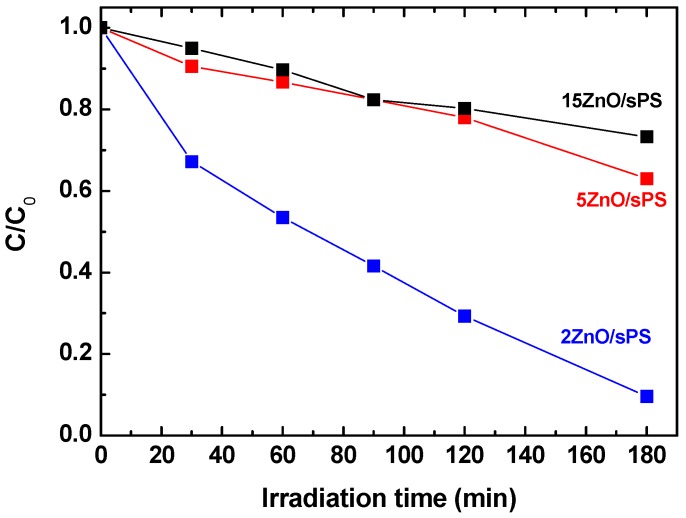
Phenol concentration in aqueous solutions treated with the ZnO/s-PS δ-form aerogels with ZnO content from 2 wt% (i.e., 0.08 g/L of ZnO) to 15 wt% (i.e., 0.6 g/L of ZnO).

The photocatalytic activity of the ZnO/s-PS δ-form aerogels, having different ZnO loadings, are reported in Figure 7.

For the 15 and 5 wt% ZnO dosages, Figure 7 clearly shows poor photocatalytic performances and, after 180 min of UV irradiation, a phenol degradation of approximately 27% and 37%, for 15 and 5 wt% ZnO, respectively.

This poor photocatalytic activity which is not associated by a lower phenol sorption capacity of the high loading aerogels (see Column 5 of Table 1) is due the formation of large size ZnO agglomerates as shown by the SEM images of Figure 6. A similar behavior has been previously observed for the Escherischia coli inactivation [24], atrazine degradation [25] as well as organic dyes removal on TiO_2_ based photocatalysts [26].

**Figure 8:**
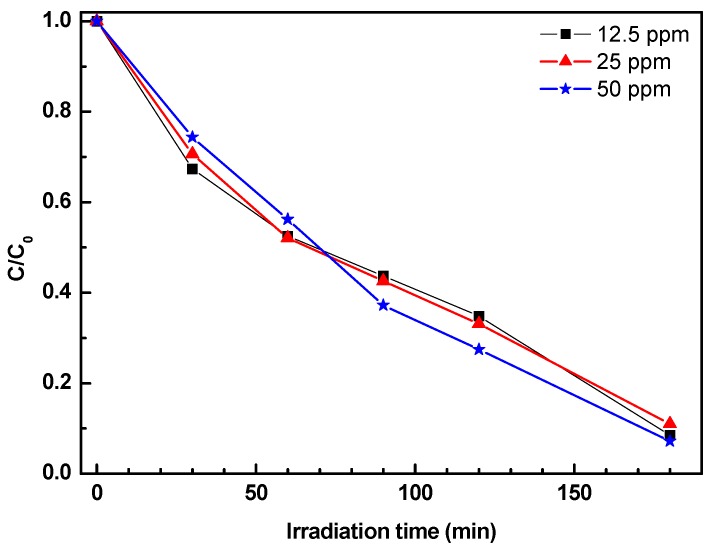
Phenol concentration in aqueous solutions, with phenol initial amount in the range 12.5–50 ppm, treated with the 2ZnO/s-PS δ-form aerogel.

The effect of the initial phenol concentration on photocatalytic efficiency was also investigated in the concentration range 12.5–50 mg/L using the 2ZnO/s-PS δ-form aerogel. The results are reported in Figure 8.

Figure 8 shows that the photocatalytic degradation rates remain constant for the range of concentrations investigated. This behavior differs from the results previously reported in literature, showing that photocatalytic degradation rate decreases with the increasing initial pollutant concentration [27,28]. Some authors [28,29] suggested that the observed worsening of photocatalytic activity was due to the competition for the adsorption sites on the photocatalyst surface between the target pollutant and the reaction by-products which increase by increasing the pollutant concentration [29].

In the ZnO/s-PS composite catalytic system described in this paper, the photocatalyst particles are dispersed in a polymeric matrix whose crystalline phase has cavities capable of competing with the catalyst surface in the reaction by-products sorption. Since the number of cavities of the polymer crystalline phase is much higher than the phenol molecules in solution, as in the tested phenol concentrations, a number of active sites on the photocatalyst surface remain always available for phenol adsorption. Therefore, phenol degradation kinetics is independent of phenol concentration itself.

**Figure 9:**
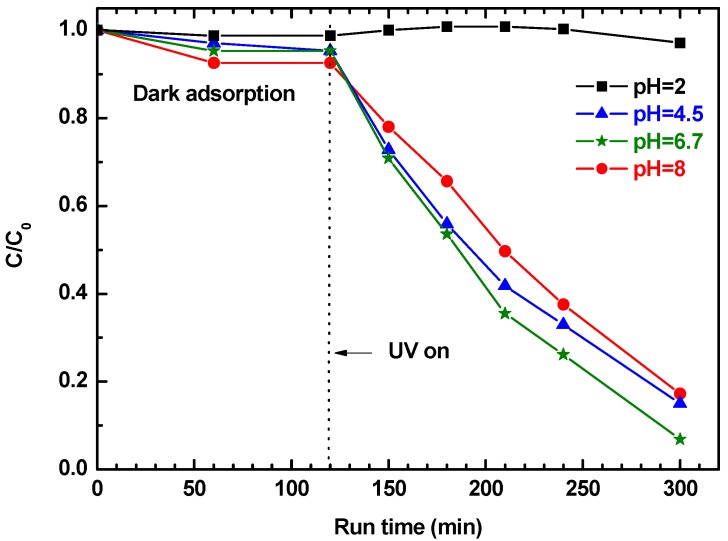
Phenol concentration in aqueous solutions, with pH in the range 2–8, treated with 2ZnO/s-PS δ-form aerogel.

The effects of pH on the photocatalytic degradation efficiency of 2ZnO/s-PS δ-form aerogel were investigated in the pH range from 2 to 8. The results are shown in Figure 9.

**Figure 10:**
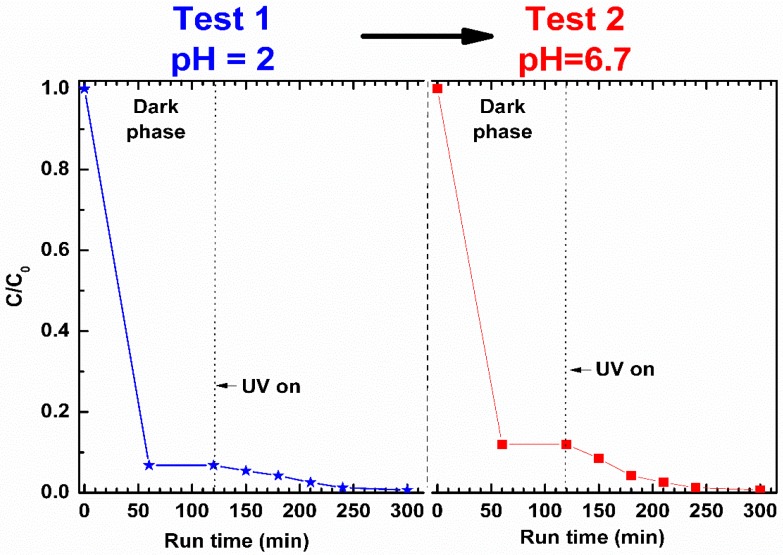
Toluene concentration in aqueous solutions, at pH = 2 (**left**) and pH = 6.7 (**right**), treated with 2ZnO/s-PS δ-form aerogel.

Figure 9 shows that the pH of the solution strongly influences phenol removal. In particular, for pH ranging from 8 to 4.5, the phenol is first absorbed in the ZnO/s-PS composite system during the dark phase, and then degraded in the UV exposure phase, while at pH = 2 phenol is neither absorbed or degraded. The pH dependence of the phenol adsorption from water is a well-known phenomenon, regardless of the adsorbent [30]. In detail, the absence of phenol adsorption obtained for the 2ZnO/s-PS aerogel at pH = 2 is in agreement with the behavior reported for different materials [31,32,33]. This behavior is generally explained by the occurrence of electrostatic repulsion between the protonated phenol (i.e., neutral form) and the partially charge surface of the absorbent. For our system, a charged aerogel surface is also conceivable. Moreover, the formation of H_3_O^+^-phenol clusters in acidic conditions, also reported in literature [34], probably contributes to the decrease of the aerogel sorption. In order to verify that the absence of photodegradation observed at pH = 2 is effectively due to the absence of phenol uptake in the polymer aerogel phase and not to the dissolution phenomena, which typically occurs in strong acidic conditions [35], two photocatalytic tests on solutions containing toluene as the pollutant model were sequentially carried out at pH = 2 and 6.7 by using 2ZnO/s-PS δ-form aerogel without any regeneration step (Figure 10).

At pH = 2 (Test 1 in Figure 10), the toluene removal by aerogel sorption in the dark phase is very high. After 60 min, approximately 93% of the toluene initially present in the solution was removed. The larger uptake of toluene (Figure 10) with respect to phenol (Figure 9) can be attributed to the strong affinity between the non-polar s-PS polymer phase and the less polar toluene (dipole moments of toluene and phenol are 0.375 and 1.224 D [36], respectively). When the UV lamps were switched on, the concentration of toluene in the aqueous solution decreased until it reached, after 300 min, the complete removal of the pollutant.

In the subsequent test (Test 2 in Figure 10) at pH = 6.7, performed on the not-regenerated sample, the toluene uptake in s-PS during the dark phase (88%) is substantially comparable to that of the test at pH = 2 (93%). This proves that the ZnO photocatalyst action at pH = 2 removed guest toluene molecules from the polymer phase allowing it to absorb again. In the UV exposure phase, the removal of toluene from the aqueous solution at pH = 6.7 is once again total, showing that the catalyst remains active even after being used in strong acidic solution.

**Figure 11:**
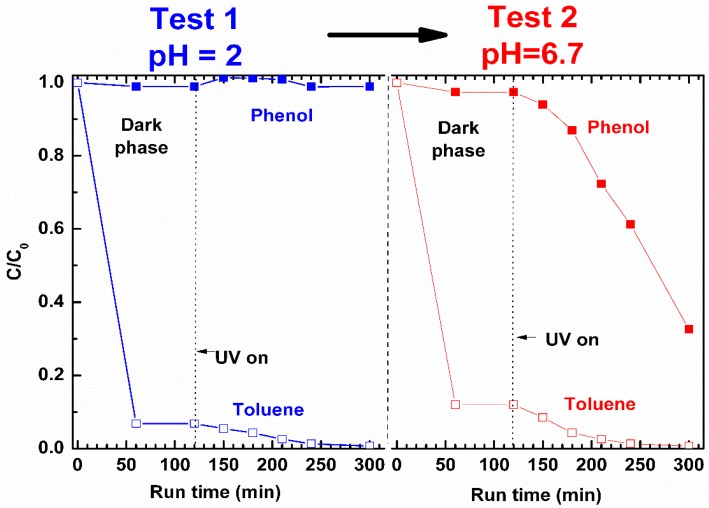
Toluene (open symbols) and phenol (closed symbols) concentration, in binary aqueous solutions at pH = 2 (**left**) and pH = 6.7 (**right**), containing 50 mg/L of both target molecules, treated with 2ZnO/s-PS δ-form aerogel.

In Figure 11, the additional photocatalytic tests on a binary aqueous solution, containing 50 mg/L of both phenol and toluene, carried out sequentially at pH = 2 and 6.7 by using 2ZnO/s-PS δ-form aerogel without any regeneration step, are also reported.

The data reported in Figure 11 show that at pH = 2 (Test 1 in Figure 11), after 300 min, toluene is completely removed from the binary solution while the degradation of protonated phenol is totally inhibited.

At pH = 6.7 (Test 2 in Figure 11), the 2ZnO/s-PS aerogel is effective in the degradation of both toluene and phenol, but the photocatalytic reaction rates are very different from each other.

After 180 min of UV irradiation, the phenol and toluene degradation were approximately 12% and 97%, respectively. It is worth noting that the degradation kinetic of toluene in aqueous binary solution is very similar to that observed in the aqueous solution of toluene alone (Figure 10). However, the phenol degradation kinetic strongly reduced in the presence of toluene (after 300 min, 70% of phenol is removed from binary solution compared to 96% removed from solution of phenol alone as shown in Figure 3). These data can be rationalized on the basis of the greater affinity of toluene with the non-polar polymer matrix (in Test 2 of Figure 11, approximately 90% of toluene is absorbed compared to 2% of phenol in the first 50 min). When the phenol and toluene molecules are absorbed in the s-PS polymer, they compete for the occupation of both the catalytic sites and the s-PS crystalline phase cavities. The greater toluene uptake in the s-PS polymer allows toluene to occupy more catalytic sites than phenol, and thus to be degraded more rapidly.

Therefore, these results demonstrate that pollutants with higher affinity towards polymer are the first ones to be selectively removed from the water solutions. Furthermore, the selectivity can be controlled by tuning the pH of the pollutant water solutions without any loss of ZnO photocatalytic efficiency.

## 4. Conclusions

The results reported in this work clearly establish that the polymeric matrix of photocatalyst/s-PS composite aerogels play a primary role on the photodegradation efficiency, since the polymer acts not only as support for the photocatalyst, but also as a pollutant pre-concentrator. In detail, it was shown that monolithic polymer aerogels presenting the s-PS nanoporous δ crystal phase can be used as a support of catalytic ZnO nanoparticles, without loss of photocatalytic efficiency, while aerogels, being in the s-PS non-porous γ crystal form, do not show any photocatalytic activity. The aerogel components have an effective synergistic action, since the s-PS polymer concentrates the pollutants into the cavities of its own crystalline phase, while the ZnO catalytic sites degrade the molecules adsorbed on them.

Consecutive tests carried out on phenol aqueous solutions using the same experimental conditions and without any aerogel regeneration, showed a complete reusability and high stability of the ZnO/s-PS composite aerogels. Moreover, the tests carried out on aqueous solutions of phenol and toluene at different pH values showed that the polymer matrix, which protects the catalyst from water, also prevents the dissolution of ZnO that typically occurs in strongly acidic or basic solutions. Finally, the comparison of the degradation kinetics of binary water solutions of phenol and toluene also showed that the nanoporous s-PS polymeric phase imparted to the ZnO/s-PS composite aerogels marked degradation selectivity towards the pollutants, such as toluene, which are highly soluble in the polymer.

In addition to the high photocatalysis efficiency, selectivity, and complete reusability without regeneration steps, monolithic ZnO/s-PS composite aerogels based on the nanoporous crystalline form of s-PS also combine the advantages of good chemical stability and excellent mechanical properties, which allow an easy recovery after water treatments.

These features, together with a consolidated manufacturing process, make the ZnO/s-PS photocatalytic aerogels extremely interesting for large-scale practical applications in the removal of POPs from water and wastewater.

## Figures and Tables

**Table 1:** ZnO dosage, aerogel apparent porosity, BET surface area of ZnO/s-PS aerogels with different polymer crystalline phases. Phenol removal in dark (after 120 min) and UV irradiation (after 300 min), obtained by using ZnO/s-PS aerogels in aqueous solutions containing 50 mg/L of phenol at PH = 6.7.

Crystalline Phase	ZnO (wt%)	Porosity (%)	BET (m^2^/g)	Phenol Removal (%)	
				Dark	UV
δ nanoporous	0	84	290	5	5
δ nanoporous	2	85	276	5	96
γ nanoporous	2	84	77	0	13
δ nanoporous	5	85	279	6	37
δ nanoporous	15	84	258	5	27

## Supplementary Materials

The following are available online at https://www.mdpi.com/2079-4991/9/11/1509/s1, Figure S1: The X-ray diffraction patterns of ZnO/s-PS aerogels with ZnO loading up to 15 wt%.
