# Supplementary material for: Highly Robust and Selective System for Water Pollutants Removal: How to Transform a Traditional Photocatalyst into a Highly Robust and Selective System for Water Pollutants Removal
Source: Nanomaterials (Basel). 2019 Oct 23;9(11):1509. doi: 10.3390/nano9111509 (PMC6915367; doi:10.3390/nano9111509)
Supplement: Supplementary file 1 [file nanomaterials-09-01509-s001.pdf]

Supplementary Materials

Highly Robust and Selective System for Water Pollutants Removal: How to Transform a Traditional Photocatalyst into a Highly Robust and Selective System for Water Pollutants Removal

Olga Sacco, Vincenzo Vaiano, Christophe Daniel, Wanda Navarra and Vincenzo Venditto

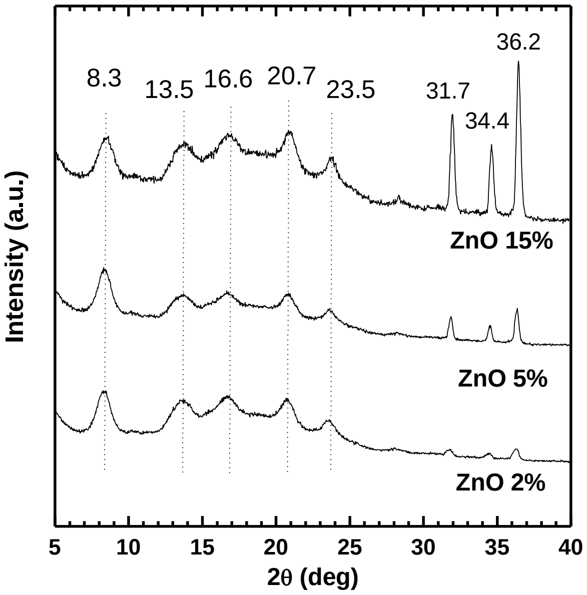

Figure S1. XRD patterns of ZnO/s-PS δ-form aerogels containing 15, 5, and 2 wt% of ZnO.
